# Supplementary material for: Mortality prediction of inpatients with NSTEMI in a premier hospital in China based on stacking model
Source: PLoS One. 2024 Dec 31;19(12):e0312448. doi: 10.1371/journal.pone.0312448 (PMC11687764; doi:10.1371/journal.pone.0312448)
Supplement: S1 File — (DOC) [file pone.0312448.s001.doc]

**Abbreations:**

CRP =C-reactive protein

TNT
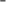
 HS=high sensitivity troponin T

CK_ MB
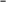
 =creatine kinase isoenzyme

NT_BNP =N-terminal brain natriuretic peptide
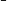


HDL
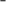
 C=High density lipoprotein cholesterol

MtoH=monocyte to high density lipoprotein ratio

MtoL=Monocyte to lymphocyte ratio

WBC=white blood cell

LYMPHRATIO=Lymphocyte ratio

NEUNUM=number of neutrophils

MONONUM=number of monocytes

HGB=hemoglobin

MCV=Mean corpuscular volume

MCHC =mean corpuscular hemoglobin concentration

RDW
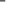
 SD=standard deviation of red blood cell distribution width

AST=glutamic oxaloacetic transaminase

TBIL=total bilirubin

IBIL=indirect bilrubin

TRIG=triglyceride

UA=blood uric acid

RVOT=right ventricular outflow tract

AOmid=mid of aortic dimension

AV=aortic valve

RV=right aterial diameter

LV=left ventricle

EDV=end diastolic volume

EF=ejection fraction

EJBKEF =Mitral orifice flow velocity peak E

EA =E peak/A peak

Syspressure=systolic blood pressure

CREA=creatinine

cTNI=cardiac troponin I

DD =D-dimer

LDL
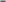
 C=Low density lipoprotein cholesterol

NtoH=Neutrophil to high density lipoprotein ratio

NtoL=Neutrophil to lymphocyte ratio

CHOL=cholesterol

NEUTRATIO=Neutrophil ratio

MONORATIO=Monocyte ratio

LYMPHNUM=number of lymphocyte

RBC=red blood cell

HCT=hematocrit

MCH=mean corpuscular hemoglobin

RDW
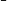
 CV=red blood cell volume distribution

ALT=glutamic pyruvic transaminase

ALB=serum albumin

DBIL=direct bilirubin

BUN=urea nitrogen

ZDB=Lipoprotein(a)

CO2=carbon dioxide

AOtop=top of aortic dimension

AObottom=bottom of aortic dimension

LA=left atrial diameter

IVS=interventricular septal thickness

LVPW=left ventricular posterior wall thickness

ESV=left ventricular end systolic volume

SV=stroke volume

EJBKAF=Mitral orifice flow velocity peak A

BMI=body mass index

Diapresure=diastolic blood pressure
